# Evaluating respiratory cryptosporidiosis in pediatric diarrheal disease: protocol for a prospective, observational study in Malawi

**DOI:** 10.1186/s12879-019-4380-x

**Published:** 2019-08-19

**Authors:** Wongani Nyangulu, Wes Van Voorhis, Pui-Ying Iroh Tam

**Affiliations:** 1 Malawi-Liverpool Wellcome Trust Clinical Research Programme, Blantyre, Malawi; 2 University of Washington, Seattle, WA USA; 3 University of Malawi College of Medicine, Blantyre, Malawi; 4 Liverpool School of Tropical Medicine, Liverpool, UK

**Keywords:** Cryptosporidiosis, *Cryptosporidium* spp., Gastrointestinal disease, Respiratory infection, Children, Prospective study

## Abstract

**Background:**

Cryptosporidium is among the most common causes of severe diarrhea in African children 0–23 months old. It is associated with excess mortality, stunting and malnutrition. The most common manifestation of cryptosporidium is intestinal diarrheal disease. However, respiratory cryptosporidiosis has been documented in up to a third of children presenting with diarrhea. It is unclear whether respiratory involvement is a transient phenomenon or a reservoir for gastrointestinal (GI) disease. This study aims to evaluate the role of respiratory cryptosporidiosis in pediatric diarrheal disease.

**Methods:**

This is a prospective, observational study conducted at Queen Elizabeth Central Hospital (QECH) in Blantyre, Malawi. Young children aged 2–24 months hospitalized with diarrhea will be enrolled. Enrolled children will have induced sputum, nasopharyngeal (NP) swab and stool samples collected. All participants positive for cryptosporidium on sputum/NP/stool PCR testing will be followed up fortnightly after discharge from the hospital up to 8 weeks post-discharge. Sputum/NP/stool sample collection will be done at each visit. The primary outcomes will be presence of Cryptosporidium spp. in sputum/NP/stool. The secondary outcome will be presence of respiratory and GI symptoms, mortality and stunting. Ethical approval was obtained from the University of Malawi College of Medicine Research Ethics Committee (COMREC) and the Liverpool School of Tropical Medicine (LSTM) research ethics committee.

**Discussion:**

The study began recruitment activities at QECH in February 2019. The protocol allows for expansion of recruitment to secondary sites within Blantyre and Chikwawa districts in the event that targets are not met at QECH. Study recruitment is expected to continue until early 2020.

## Background

Cryptosporidiosis is a cause of severe diarrhea [1], excess mortality [2, 3], and stunting, and is associated with malnutrition [4]. The Global Enteric Study (GEMS) identified Cryptosporidium as the second most common cause of diarrhea among infants (0–11 months) in all four African countries studied (The Gambia, Mali, Mozambique and Kenya) regardless of HIV prevalence, and among the top five causes for older children (12–23 months) [2]. The Etiology, Risk Factors and Interactions of Enteric Infections and Malnutrition and the Consequences for Child Health and Development Program (MAL-ED) noted that cryptosporidiosis is the fifth highest attributable pathogen in all pediatric diarrhea in the community setting, and has increased frequency among those with prolonged and severe diarrhea [1]. Cryptosporidiosis is associated with excess mortality in children who had the infection in infancy, and this excess mortality persists into the second year of life. Respiratory cryptosporidiosis has been documented in up to a third of children presenting with diarrhea [5]; furthermore, respiratory detection without intestinal involvement has been reported, raising the possibility of primary respiratory infection with *Cryptosporidium* spp., either by inhalation or by contact with fomites [6].

Malawi has a high prevalence of Cryptosporidium. In a recent case control study of children who presented with diarrhea (≥ 3 loose stools within a 24 h period) to QECH, *Cryptosporidium* spp. (27.8%) was the third most common pathogen detected behind rotavirus (34.7%) and adenovirus (29.1%) among cases [7]. This is much higher than previous reports likely due to improved diagnosis using molecular methods (PCR) [7, 8]. The rainy season is associated with higher incidence of disease compared to the dry season but a study comparing Blantyre (urban) to Chikwawa (rural) did not show a significant difference in number of cases between urban and rural dwellers [8].

### Rationale

Studies have identified *Cryptosporidium* spp. in sputum but have not looked at whether respiratory involvement is a transient phenomenon or a reservoir for gastrointestinal (GI) disease. Our study is unique in that this is the first longitudinal study to evaluate for pulmonary and GI Cryptosporidium concurrently. If respiratory cryptosporidiosis is established to be a reservoir for diarrheal disease, findings would inform the development of systemic therapeutic interventions against Cryptosporidium.

The main study hypothesis is that pulmonary Cryptosporidium can be a nidus of infection for Cryptosporidium. Secondly, we hypothesize that pulmonary and GI Cryptosporidium will lead to worse outcomes (mortality, stunting) than those with only GI Cryptosporidium.

## Study objectives

### Primary objectives

The primary objectives are: (1) To estimate the proportion of young children on admission hospitalized with Cryptosporidium diarrhea that have Cryptosporidium detected from the respiratory tract; and (2) To evaluate the longitudinal pattern of pulmonary cryptosporidiosis in children hospitalized with Cryptosporidium diarrhea.

### Secondary objectives

The secondary objectives are: (1) To identify risk factors for respiratory cryptosporidiosis in children with diarrhea; (2) To determine the proportion of children with diarrhea and with Cryptosporidium in the respiratory tract that have associated respiratory symptoms; and (3) To determine the detection rate of Cryptosporidium in the GI and respiratory tract, and longitudinally, detection in one tract or the other and the relationship to respiratory and GI symptoms.

### Primary outcomes

The presence of *Cryptosporidium* spp. in sputum/NP swab and stool.

### Secondary outcomes

The presence of respiratory and GI symptoms, mortality and stunting.

## Methods/design

### Study type

This prospective, observational and longitudinal study will evaluate the role of respiratory cryptosporidiosis in pediatric diarrheal disease.

### Study location

The primary site for this study is QECH located in Blantyre, Malawi. Subjects will be recruited from the Pediatric Accident and Emergency and pediatric wards at QECH. If recruitment targets are not met at QECH, recruitment will be expanded to secondary sites including Blantyre District Health Office health centers and Chikwawa District Hospital in Chikwawa, Malawi.

### Study population

Young children 2–24 months of age hospitalized with a primary diagnosis of diarrhea will be enrolled into the study, provided parents/ guardians complete the informed consent process and children fulfill all the inclusion and exclusion criteria.

*Inclusion criteria*

- Male or female
- Age 2–24 months
- Hospitalized with diarrhea (defined as 3 or more loose stools within 24 h)
- Lives within 15 km radius of Blantyre district
- Positive on sputum/NP swab or stool for Cryptosporidium by PCR
- Parent/guardian consent for enrollment

*Exclusion criteria*

- Dysentery (visible blood in loose stool)

### Study procedures

#### Recruitment

Persons screened for study entry will be those presenting with primary GI symptoms to QECH and/or health centers in Blantyre or Chikwawa region, and who are eligible for hospitalization. Eligible patients meet all the inclusion and none of the exclusion criteria.

Subjects for this study will be recruited from the study site by word of mouth. Patients attending clinics other than health centers or district hospitals with inpatient facilities will be informed about the study at Triage. Before any study related activities, the study team will obtain informed consent.

#### Screening

Following admission and after obtaining informed consent, an electronic folder will be created on tablet computers for potential participants. Data collected will include demographic information, medical history, physical examination and results of relevant investigations performed as part of routine care (including full blood count (FBC), urea and electrolytes (U&E), and liver function tests (LFTs), malaria parasite screen, blood and/or CSF culture, TB GeneXpert and acid fast bacilli (AFB) testing). All HIV exposed infants will have HIV DNA PCR done.

All enrolled patients will have induced sputum/NP swab and stool samples collected. Induced sputum will only be collected if there are no contraindications (based on PERCH criteria for induced sputum collection): severe hypoxia < 92% on supplemental oxygen; inability to protect airways; severe bronchospasm at admission (defined as continued hypoxia < 92% after appropriate bronchodilator therapy, with other markers of respiratory distress); seizure within the past 24 h; deemed inappropriate by the clinician for another reason (e.g. midface trauma, inhalational injury, pulmonary effusion, congestive heart failure, congenital heart disease, etc.). If the above symptoms/conditions resolve during the hospital course, induced sputum collection may be reconsidered at that point.

#### Sampling procedures

The induced sputum procedure will be as follows. Subjects will be given a nebulized β-agonist (salbutamol) followed by 3% sodium chloride mist to inhale for 5–15 min. A sterile catheter will then be passed through the nose into the posterior nasopharynx and suction applied to aspirate contents. If the child does not tolerate the procedure or has an adverse reaction, the procedure will be discontinued and the child will be monitored up to 4 h post procedure. Once stabilized, and parents are amenable, the induced sputum collection can be attempted 24 h later.

Quality of sputum will be documented by microscopy (good quality: ≤10 squamous epithelial cells/low powered field). The first 100 subjects screened will also have NP swab collected in addition to induced sputum, in order to compare the yield of Cryptosporidium detection between these two specimen types. If the yield is deemed equivalent, subsequent respiratory tract sampling after the first 100 subjects will include only NP swabs. All sputum and stool specimens from recruited subjects will also be subjected to multiplex testing for multiple pathogens but the results will not be returned by enrollment.

If the patient remains hospitalized for longer than a week then sputum/NP swabs and stool samples will be collected weekly until discharge. With each sample collection, the patients will be assessed for symptoms and results from relevant laboratory investigations performed as part of routine care will be recorded. At discharge, the patients will be assessed for symptoms but no specimens will be collected.

#### Enrollment and follow-up

Participants that are positive for Cryptosporidium on NP/sputum/stool testing will continue to be followed up in the study (Table 1). They will be instructed to return to the clinic for a follow-up visit fortnightly for evaluation of symptoms, physical assessment, as well as sputum/NP and stool sampling. A final follow up visit will be scheduled approximately 8 weeks after discharge for final evaluation of symptoms, physical assessment, and sputum/NP and stool sampling. Table 2 provides an overview of all study visits and scheduled study events.

**Table 1:** Overview of study visits

Visit	Activities
1	Admission to the inpatient ward, assessment, collection of induced sputum and stool sample
	1a. If sputum/NP/stool is positive for *Cryptosporidium,* continue to follow
	1b. If sputum/NP/stool is negative for *Cryptosporidium,* discontinue from study
	If the patient remains hospitalized for longer than 2 weeks then sputum/NP swab and stool samples will be collected fortnightly until discharge.
2	Outpatient Week 2 follow-up visit, assessment, collection of induced sputum/NP swab and stool sample
3	Outpatient Week 4 follow-up visit, assessment, collection of induced sputum/NP swab and stool sample
4	Outpatient Week 6 follow-up visit, assessment, collection of induced sputum/NP swab and stool sample
5	Outpatient Week 8 follow-up visit, assessment, collection of induced sputum/NP swab and stool sample

**Table 2:** Summary of study events schedule

Study event	Study day/Interval						
	Inpatient			Outpatient visits (Week post-discharge)			
	Admission	Every 2 weeks	Discharge	2	4	6	8
Informed consent	X						
Inclusion/exclusion criteria	X						
Demographics	X						
Medical history	X	X	X	X	X	X	X
Vital signs	X	X	X	X	X	X	X
Physical exam	X	X	X	X	X	X	X
HIV rapid test +/− HIV DNA PCR^a^	X						
Relevant investigations (e.g. CXR, FBC, blood culture, etc.)	+/− Only if clinically indicated						
Induced sputum for microscopy, *Cryptosporidium* qPCR, and multiplex testing	X	X		X^b^	X^b^	X^b^	X^b^
NP swab for *Cryptosporidium* qPCR for first 100 subjects only	X	X		X^b^	X^b^	X^b^	X^b^
Stool for TaqMan array	X	X		X^b^	X^b^	X^b^	X^b^

^a^Will be performed on all infants < 18 months who are positive on rapid HIV test

^b^Subset of those positive on sputum/NP swab or stool for *Cryptosporidium* PCR on admission

### Laboratory processing

#### HIV testing

All participating children will be tested for HIV on admission by QECH HIV testing counselors. These are part of routine care and so will enhance the management of these individuals. In line with national guidelines, HIV testing will be with 2 complementary rapid tests (UniGold™ and Determine™) according to manufacturers’ instructions. As HIV antibody testing is not a reliable marker of HIV status in infants until about 18 months of age, we will perform HIV DNA PCR testing on all recruited children < 18 months with a positive HIV test.

#### Induced sputum and stool processing

Induced sputum collection will be done in a well-ventilated room (at QECH, this will be a patient procedure room in the Paediatric Research Ward) equipped with an emergency tray. Quality of the sputum will be documented by microscopy. All participating children will have admission sputum and stool specimens evaluated for Cryptosporidium*,* by Cryptosporidium PCR for sputum or the TaqMan assay for stool, in batched testing. Only the admission specimens that are positive for Cryptosporidium in either sputum or stool will have all subsequent specimens processed for multiplex testing. Multiplex testing on sputum will test for bacteria (*S. pneumoniae, S. aureus, M. catarrhalis, B. pertussis, H. influenzae* and *H. influenzae* type b, *C. pneumoniae, M. pneumoniae, K. pneumoniae, L. pneumophila,* and Salmonella species); viruses (influenza A/B/C, RSV A/B, parainfluenza virus types 1–4, coronaviruses NL63, 229E, OC43, and HKU1, human metapneumovirus A/B, rhinovirus, adenovirus, enterovirus, parechovirus, bocavirus, cytomegalovirus); and parasites (*P. jirovecii*). Multiplex testing of stool will test for viruses (rotavirus, norovirus GII, adenovirus, astrovirus, sapovirus), bacteria (enterotoxigenic *Escherichia coli* (ETEC), enteropathogenic *E. coli* (EPEC), enteroaggregative *E. coli* (EAEC), Shiga-toxigenic *E. coli* (STEC), Shigella/enteroinvasive *E. coli*, *Salmonella*, *Campylobacter jejuni*, *C. coli*, *Vibrio cholerae*, *Clostridium difficile*), and parasites (*Cryptosporidium*, *Giardia lamblia*, *Entamoeba histolytica*, *Ascaris lumbricoides*, and *Trichuris trichiura*).

### Statistical analysis

#### Sample size

About 250 subjects will be screened. Various studies identified *Cryptosporidium* diarrhea in 12.5–13.8% of children [2, 5]. Therefore, with a sample size of 250 children, we anticipate 30–35 to have positive stool samples at admission.

#### Analysis

The primary endpoints (detection of Cryptosporidium in stool, sputum) are binary. Frequencies and proportions of observed levels will be reported for binary and categorical variables, with exact binomial 95% confidence intervals given where appropriate. For all continuous variables we will report the population size, the non-missing sample size, the mean, and standard deviation, median, minimum, maximum and interquartile range. Age distribution will be reported as a categorical variable with age bands defined as 2–11 months and 12–24 months.

Based on a planned sample size of 250 children, to estimate proportions we expect to achieve the absolute margins of error given in Table 3 for different values of proportions and sample sizes of children hospitalized with Cryptosporidium diarrhea.

**Table 3:** Absolute margins of error for different values of proportions and sample sizes of children hospitalized with Cryptosporidium diarrhea, based on a sample size of 250

Proportion of children with Crypto diarrhea (%)	Number of children with Crypto diarrhea	Proportion of children hospitalized with Cryptosporidium diarrhea with a positive sputum test (%)							
		15	20	25	30	35	40	45	50
10	25	15.77	16.94	17.89	19.28	19.75	20.10	20.33	20.45
12	30	13.48	15.43	16.80	17.33	17.76	18.37	18.67	18.70
14	35	12.73	14.25	15.38	16.22	16.54	17.01	17.26	17.31
16	40	12.06	13.30	14.25	14.98	15.53	15.90	16.13	16.20

For Objective 1a, based on the Mor et al. paper we expect 14.7–35.4% of recruited subjects with diarrhea to have *Cryptosporidium* in stool and sputum at admission (4–12 sputum-positive out of a total of 35 stool-positive).

For Objective 1b, we expect the proportion to be higher. If the estimated proportion is 35% and we estimate this proportion from *n* = 30 children hospitalized with *Cryptosporidium* diarrhea, then we can estimate this proportion with an absolute margin of error of 18% (exact binomial 95% confidence interval [17, 53%]).

For Objective 2, we do not have an expected estimate as our study will be the first to look at this. However, Table 3 gives a range of proportions between 15 and 50%.

Time to subsequent detection in the respiratory tract or GI tract will be evaluated using time-to-event analysis methods for interval-censored data. Specifically, we will use non-parametric maximum likelihood estimators and generalized log-rank tests for unadjusted analyses and Cox regression for adjusted analyses.

Comparisons between groups of patients (defined by age band, HIV status, etc.) will be performed using the exact Fisher test for binary and categorical variables, t-test (two groups) or ANOVA (three or more groups) for approximately normally distributed variables and Wilcoxon rank sum (2 groups) or Kruskal-Wallis (3 or more groups) tests for variables with severely non-normal distributions. To evaluate outcomes longitudinally we will use mixed models for data with patient ID treated as a random factor.

### Data safety and monitoring

Data will be collected onto standard proformas and all records will be held securely in MLW project offices. Proformas will be destroyed after 10 years. Each study participant will be assigned a unique study number at enrolment. Demographics and relevant medical history will be collected on a structured case record form (CRF) that will be electronically captured (ODK system) and stored on a password-protected database. Data files (anonymized) will be exported to Stata (Statacorp, USA) for analysis.

All data will be acquired and validated by the MLW data team. All databases will be protected by passwords. The only sources of person-identifiable information will be the consent form, enrollment log, and the participant tracking form. The latter will detail the subjects’ name, contact details and the unique study number, in order to provide a link with the anonymized clinical data. The paper copy will be kept in a secured locked cabinet held separately from the CRF hard copies. The electronic copy will be stored in a password-protected database. Only authorized study personnel will have access to this information.

The study will be monitored by an internal monitoring committee, Malawi-Liverpool Wellcome Trust (MLW) Clinical Research Support Unit (CRSU) and the University of Malawi College of Medicine Research and Ethics Committee (COMREC).

### Ethical considerations

This study protocol and informed consent documents were reviewed and approved by COMREC protocol number: P.07/18/2438 and the Liverpool School of Tropical Medicine Research Ethics Committee (LSTM REC protocol number: 18–066). For the purposes of this study, COMREC will be considered the local IRB and LSTM REC the remote IRB.

#### Recruitment

Subjects for this study will be recruited from the study site by word of mouth. Patients attending clinics other than health centers or district hospitals with inpatient facilities will be informed about the study at triage. A poster will be placed at each clinic announcing the study. All study procedures will be performed at QECH, health centers or district hospitals with inpatient facilities.

After meeting inclusion/exclusion criteria and providing documented informed consent forms (ICF), subjects will be screened using a PCR for the presence of Cryptosporidium infection. Subjects testing positive for Cryptosporidium infection will continue participation in the longitudinal phase of the study. The longitudinal phase of the study will be explained and discussed with the subject at the time of enrollment, and will be reviewed with the patient if they test positive.

#### Informed consent process

Young children will be recruited. Before any study-related activities and in agreement with applicable regulatory requirements, the Investigator will ensure that the subject and parent/guardian is fully informed about the aims, procedures, potential risks, and potential benefits of the study. The subject’s parent/guardian will be given the written, IRB-approved ICF, allowed ample time to read the consent form, allowed to ask questions about the study, have their questions answered and then be given time to decide if s/he would like her child to participate in the study. It will be emphasized that participation is voluntary, and that the subject has the right to withdraw from the study at any time without prejudice.

For enrollment into the study, the subject’s parent/guardian will need to sign an ICF. The Investigator will obtain the parent/guardian’s voluntary, personally signed and dated ICF before any study-related procedure can be conducted. The original, signed ICF(s) will be kept in the site study file. Subjects volunteering to participate in this study will be compensated for their time according to the terms specified by COMREC. Specifically, they will be given the equivalent of USD 10 in Malawi Kwacha to cover transport expenses when asked to visit the hospital for study-related procedures when participating in the specimen collection segment of the protocol, and additional payment for their time when admitted in the hospital for study-related procedures and for transport expenses for the five follow up visits.

Parents or guardians will be given written, IRB-approved informed consent forms in English or Chichewa, allowed ample time to read the consent form, allowed to ask questions about the study, have their questions answered and given time to decide if s/he would like their child to participate in the study. The informed consent will be read verbally in Chichewa to those who are unable to read. This will be done in the presence of an impartial witness who will also sign the consent form. After reading or listening, the potential participant is asked to sign the informed consent form. For those who cannot write, a thumbprint will be placed on the form with the signature of the witness and date. After obtaining informed consent, participants will be given a signed copy of the informed consent document and assigned a screening identification number.

### Risk/benefit

This study is primarily a longitudinal observational study. The main risks to the subject will be during specimen collection of blood, stool, induced sputum, and NP swabs. Good phlebotomy practices will be performed during blood draws, to minimize the risk to the subject. Both stool and NP swab collection are noninvasive procedures, routinely performed by study team members and are of minimal inconvenience for the participant.

Induced sputum collection is a standard method of specimen collection for investigating respiratory Cryptosporidium species infections, and is a much less invasive technique than other airway sampling techniques such as bronchoscopy. Sputum induction is viewed as a safe and tolerable procedure among children with respiratory issues such as asthma, with few or minor transient adverse effects, such as sore throat. Studies have evaluated sputum induction among pediatric patients who have severe or very severe pneumonia. In > 1000 procedures performed in children with severe pneumonia admitted to Kilifi District Hospital in Kenya, only 1 serious adverse event occurred (a child with a known seizure disorder had a brief convulsion during the administration of hypertonic saline; the procedure was stopped and the child recovered without sequelae [9]. Of 108 children enrolled into the pilot study in New Caledonia, none had an adverse event sufficiently severe to require increased oxygen therapy or nebulization or that led to termination of the procedure [9]. The Pneumonia Etiology Research in Child Health (PERCH) conducted induced sputum on all enrolled children between 1 and 59 months of age at 5 African sites. Induced sputum was successfully obtained on the vast majority of children at all study sites and with the correct training and resources was regarded as relatively straightforward and safe [10].

For this study, appropriate training and resources will be provided to ensure safety of the subjects. We will use the same contraindications as detailed in PERCH, even though their study was focused on severe and very severe pneumonia; we expect the majority of our enrolled patients to have diarrhoeal issues first and foremost, with any respiratory involvement expected to be minor. We have been provided space in a well ventilated room in the Pediatric Research Ward for the conduct of this procedure, which will be stocked with an emergency tray. Subjects will be monitored closely for expected AEs during this study, and appropriate clinical responses made if they occur. If recruitment is expanded to include health clinics in Blantyre district, this would be for the purpose of identifying subjects who would then be referred to QECH for consent and specimen collection.

There is no direct benefit to subjects in this study anticipated. Research on children and HIV infected adults is justified by the burden of diarrhoeal and respiratory tract infection in these populations. These data will inform ongoing management strategies, and the study participants will stand to benefit from the research findings. It is hoped that better understanding of the role of respiratory cryptosporidiosis in pediatric diarrheal disease will inform development of therapeutics. Cryptosporidium is an important cause of morbidity and mortality in malnourished young children in the developing world and persons with HIV infection. No effective treatments are currently available for these populations. If an effective drug could be found, that would be of great benefit to society. The principal benefit of this study is indirect, potential benefit to society. However, patients will incur no cost for participation in this study. Financial reimbursement will be provided according to existing Malawi national research guidelines.

#### Dissemination of results

Results will be presented to the QECH Department of Pediatrics research meeting, and may be presented at local/regional/international conferences and published in a peer-reviewed journal.

## Discussion

It is widely accepted that the gastrointestinal tract is the principal site for cryptosporidium infection and disease. Apart from inhalation or contact with fomites, respiratory infection may result from aspiration of intestinal contents in patients with diarrheal disease or via hematogenous spread through the intestinal vasculature [6, 7]. It is unclear however, if initial infection in patients with diarrheal disease occurs first in the respiratory tract with subsequent spread to the gut or vice versa. This study is specifically designed to address this important question.

### Study status

The study began recruitment in February 2019. At the beginning of May 2019, 52 patients had been screened with 14 patients PCR positive for cryptosporidium in stool or sputum, a higher than expected recruitment return at this stage of the study. We attribute this to higher local prevalence of cryptosporidium (27.8%) compared to other studies of cryptosporidium in children (12.5–13.8%) [7, 11]. We expect a drop-off in screening and recruitment as the rainy season ends with a resurgence in the next rainy season.

### Participant safety

Fifty-one of the fifty-two enrolled participants have undergone induced sputum collection. None had contraindications to the procedure at initial contact. No adverse events or severe adverse events have been detected or reported so far. A sub study evaluating safety of induced sputum collection in Malawian children is also being conducted. This will further add to evidence on safety of induced sputum collection and optimum follow up time after the procedure, data essential to adoption of induced sputum collection in non-research settings (public hospitals).

## Data Availability

The datasets used and/or analyzed during the current study are available from the corresponding author on reasonable request.

## Abbreviations

HIV: Human immunodeficiency virus
LSTM: Liverpool School of Tropical Medicine
PCR: Polymerase chain reaction
QECH: Queen Elizabeth Central Hospital
